# Safety Results for Geographic Atrophy Associated with Age-Related Macular Degeneration Using Subretinal Cord Blood Platelet-Rich Plasma

**DOI:** 10.1016/j.xops.2024.100476

**Published:** 2024-01-24

**Authors:** Stanislao Rizzo, Maria Cristina Savastano, Benedetto Falsini, Patrizio Bernardinelli, Francesco Boselli, Umberto De Vico, Matteo Mario Carlà, Federico Giannuzzi, Claudia Fossataro, Gloria Gambini, Emanuele Crincoli, Silvia Ferrara, Matteo Ripa, Raphael Killian, Clara Rizzo, Caterina Giovanna Valentini, Nicoletta Orlando, Giorgio Placidi, Luciana Teofili, Alfonso Savastano

**Affiliations:** 1 Ophthalmology Unit, Fondazione Policlinico Universitario A. Gemelli IRCCS, Rome, Italy; 2 Catholic University “Sacro Cuore”, Rome, Italy; 3 Consiglio Nazionale delle Ricerche (CNR), Istituto di Neuroscienze, Pisa, Italy; 4 Department of Ophthalmology, Centre Hospitalier Intercommunal del Crèteil, France; 5 Department of Ophthalmology, William Harvey Hospital, East Kent Hospital University NHS Foundation Trust, Ashford, United Kingdom; 6 Ophthalmology Unit, University of Verona, Verona, Italy; 7 Ophthalmology, Department of Surgical, Medical and Molecular Pathology and Critical Care Medicine, University of Pisa, Pisa, Italy; 8 Department of Diagnostic Imaging, Oncological Radiotherapy and Hematology, Fondazione Policlinico Universitario A. Gemelli IRCCS, Rome, Italy

**Keywords:** Age-related macular degeneration, Macular degeneration, Vitreoretinal surgery, Macular surgery, Cord blood platelet- rich plasma

## Abstract

**Purpose:**

To evaluate the safety of subretinal injection of cord blood platelet-rich plasma (CB-PRP) and its possible effect in eyes affected by geographic atrophy (GA) associated with dry age-related macular degeneration (d-AMD).

**Design:**

Interventional, open-label study started in January 2021 with follow-up at 12 months (the Si.Cord Study). This study was a single-center, nonrandomized, sequential-assigned clinical trial conducted in Rome, Italy, at Fondazione Policlinico Universitario Agostino Gemelli IRCCS (ClinicalTrials.gov NCT04636853*)*.

**Participants:**

Thirteen patients (26 eyes) with bilateral d-AMD-related GA were enrolled. One eye from each patient (with more advanced GA) underwent CB-PRP treatment, and the fellow eye was considered the control. All patients participated in follow-up at 12 months.

**Intervention:**

All 13 eyes received 23-gauge (G) vitrectomy and subretinal injection of CB-PRP using a 41-gauge needle.

**Main Outcomes and Measures:**

Best-corrected visual acuity **(**BCVA) with ETDRS letters, central macular thickness using OCT, and atrophic area measured on en face OCT images were assessed at baseline, 1, 3, 6, and 12 months.

**Results:**

The BCVA in the treated group was 34.46 ± 20.8 ETDRS at baseline, 40.84 ± 20.52 at 1 month, 40.07 ± 20.34 at 3 months, 39.38 ± 19.84 at 6 months, and 35.84 ± 18.38 at 12 months. In the untreated group*,* the BCVA was 53 ± 21.1 ETDRS letters at baseline, 51.54 ± 20.99 at 1 month, 46.62 ± 19.47 at 3 months, 46.85 ± 18.58 at 6 months, and 43.92 ± 17.97 at 12 months (2-way analysis of variance: interaction of treatment by eye or time, *P =* 0.084). Central macular thickness did not show a significant intereye difference at 12 months (*P =* 0.97). The atrophic geographic areas tended to increase in both treated and fellow eyes at 12 months (*P <* 0.0001). No inflammatory reaction, endophthalmitis, retinal detachment, uveitis, or other complications due to the subretinal injection of CB-PRP were observed during the follow-up.

**Conclusions:**

Subretinal injection of CB-PRP could be safely used for d-AMD in its GA form. Despite its safety, a larger cohort of patients, and probably a new way of administration, will be needed to understand whether the CB-PRP could have a role in the GA treatment.

**Financial Disclosure(s):**

Proprietary or commercial disclosure may be found in the Footnotes and Disclosures at the end of this article.

Geographic atrophy (GA) is the advanced stage of dry age-related macular degeneration (d-AMD) and is characterized by a degenerative condition of the macula, with a demarcated area of retinal pigment epithelium (RPE)/photoreceptor/choriocapillaris atrophy in the posterior pole resulting in severe and progressive central visual loss.^1^^,^^2^ An estimation of the prevalence of GA in 5 million people worldwide was recently reported in the United States.^3^

Treatment of GA in d-AMD is currently a challenge. Many attempts have been made to slow the progression of rod and cone photoreceptor degeneration.^4^ In the last few years, researchers have focused on the ability of complement 3 (C3) inhibitors to reduce the expansion of GA lesions.^5^ Indeed, the FILLY trial recently showed that intravitreal pegcetacoplan (C3 inhibitor) dosed monthly was able to reduce the rate of GA lesion growth by 29% compared with sham treatment. Furthermore, eyes receiving intravitreal pegcetacoplan showed slower rates of progression from RPE and outer retinal atrophy than controls.^6^ DERBY and OAKS are phase 3, multicenter, randomized, double-masked, sham-controlled studies comparing the efficacy and safety of intravitreal pegcetacoplan with sham injections in patients with GA secondary to d-AMD. As reported during the 22^nd^ Euretina Congress meeting (Hamburg, Germany), pegcetacoplan seems to be effective in slowing atrophic area enlargement. In fact, DERBY showed a decrease in the atrophic area enlargement by 12% (every other month injection regimen) and 13% (monthly injection regimen) at 18 months compared with sham. OAKS showed a decrease in the atrophic area enlargement by 16% (every other month injection regimen) and 22% (monthly injection regimen) at 18 months compared with sham. Just recently (February 17, 2023, and August 4, 2023), the Food and Drug Administration approved intravitreal pegcetacoplan and avacincaptad pegol injections, respectively, as the first treatments for GA, marking a milestone in the d-AMD treatment armamentarium.^7^^,^^8^

Neuroprotective growth factors are indeed thought to have a therapeutic role in treating GA.^4^^,^^9^

Platelets are a natural reservoir of growth factors for several cell lineages. Their activation at sites of tissue injury promotes cell proliferation and tissue repair, including revascularization. Due to its effect on tissue repair, the use of autologous platelet-rich plasma (PRP) has become widely popular.^10^, ^11^, ^12^ Cord blood platelet-rich plasma (CB-PRP), the application of which in regenerative ophthalmic medicine is widely recognized, represents an important standardized source of multiple growth factors and cytokines. Furthermore, CB-PRP, as a jellified or liquid derivative, has shown specific characteristics that promote cell differentiation and proliferation and healing of damaged tissues. Clinically, these derivatives have been widely used to treat mucocutaneous lesions and eye diseases.^13^ The properties of CB-PRP suggest its use as a neuroprotective strategy in the degeneration of rod and cone photoreceptors associated with GA in d-AMD. Interestingly, CB-PRP could provide, as recently demonstrated in preclinical studies, an appropriate number of neurotrophic factors (i.e., nerve growth factor, fibroblastic growth factor) to the cones, favoring their survival.^14^^,^^15^ Because of all these potentially regenerative and antioxidant qualities, we believed that CB-PRP could be helpful in d-AMD.^16^

The purpose of this study was to assess the safety and a possible sign of efficacy of CB-PRP subretinal injection in supporting the survival of macular photoreceptors in patients’ eyes affected by GA in d-AMD.

## Methods

This study was a case-control, interventional, open-label (no masking) study started in January 2021 with a minimum follow-up of 12 months (Si.Cord Study). It was a nonprofit, single-center, nonrandomized, sequential-assigned clinical trial conducted in Rome, Italy, at Fondazione Policlinico Universitario Agostino Gemelli IRCCS, in compliance with the Declaration of Helsinki. The study protocol was submitted to and approved by the institutional Ethical Committee (ID 3417). All participants provided specific written informed consent to participate in the study after the goals and procedures were fully explained. Due to the nonprofit nature of the Si.Cord Study, it was covered by specific medical insurance, as recommended by our Ethical Committee.

### Study Population

**Figure 1:**
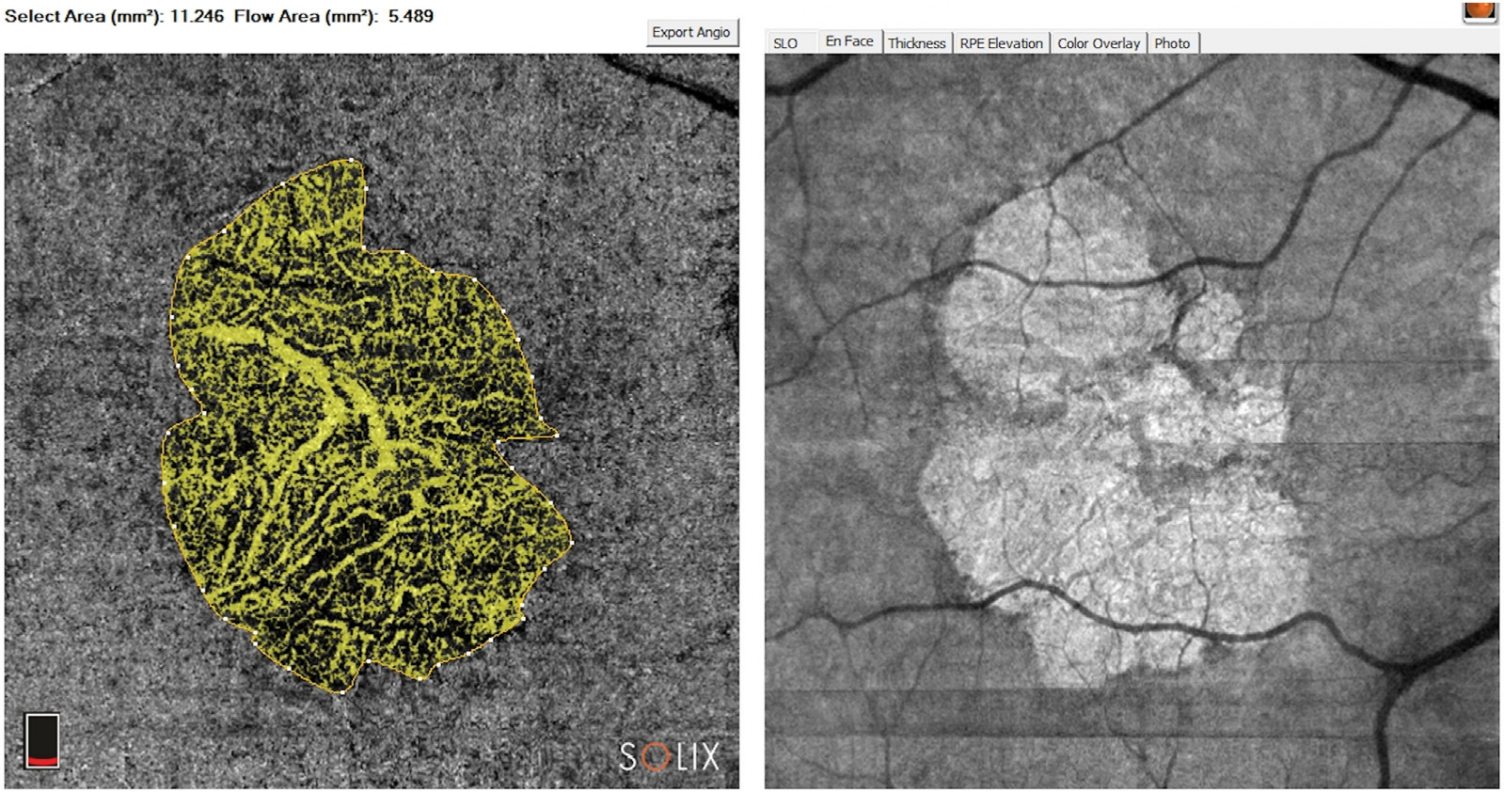
OCT angiography (OCTA) and OCT en face: dry age-related macular degeneration (geographic) of 1 patient’s treated eye (3 months' follow-up). The image shows OCTA and OCT en face for the atrophic area calculation. Atrophy assessment on en face OCT obtained by OCTA acquisition showed significant details. Atrophy selected area is 11.25 mm^2^.

A group of 13 patients’ eyes affected by d-AMD (geographic type) were examined and enrolled (Fig 1). The inclusion criteria were a diagnosis of GA in the context of d-AMD, best-corrected visual acuity (BCVA) at more than light perception, clear optical media, and no concomitant ocular or systemic diseases. The exclusion criteria were aged < 55 years, previous inflammatory and/or infective ocular diseases, presence of cataracts, and phacoemulsification surgery for cataracts performed within the previous 6 months.

Other exclusion criteria were media opacities and concomitant eye and systemic diseases (i.e., glaucoma, diabetic retinopathy, macular edema, etc.) to reduce bias in the results. All eyes underwent OCT angiography (OCTA) analysis before enrollment. The presence of macular neovascularization in either or both eyes was considered an exclusion criterion. All patients participated in the follow-up at 12 months.

### Study Intervention

**Figure 2:**
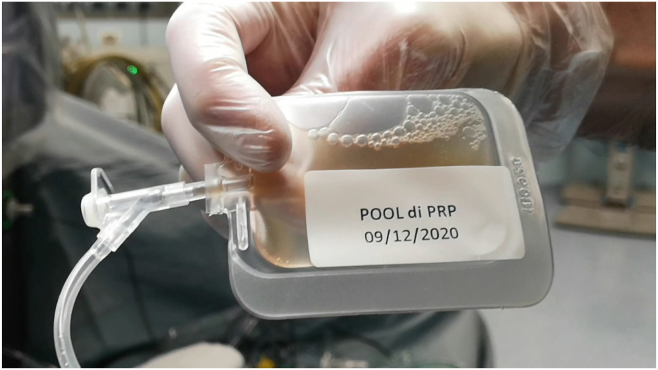
Defrosted 1-ml cord blood platelet-rich plasma (CB-PRP) sterile aliquot ready to be used.

After patient screening and consent, the patients underwent vitrectomy and a subretinal injection of CB-PRP provided by the Cord CCB Bank of Fondazione Policlinico Universitario Agostino Gemelli IRCCS, Rome, Italy, according to the procedures provided by the current legislation on blood components for nontransfusion use. All patients were treated in the worst eye, and fellow eyes were used as controls. Cord blood PRP is a blood product for nontransfusion use produced according to standardized procedures defined by the Italian regulation on blood product (Decree of the Ministry of Health [November 2, 2015]: Provisions relating to the quality and safety requirements of blood and blood components. ***Gazzetta Ufficiale*** n. 300 (December 28, 2015); Decree of the Ministry of Health (August 19, 2019): Amendments to the decree of November 2, 2015, containing: "Provisions relating to the quality and safety requirements of blood and blood components". ***Gazzetta Ufficiale*** n. 226 [September 26, 2019]). The source material for PRP manufacturing consists of cord blood units collected at the Cord Blood Bank of the Fondazione Policlinico Gemelli IRCCS. These units are allogeneic solidary donations for hematological patient candidates for hematopoietic stem cell transplantation. In this field, the use of cord blood represents a therapeutic practice consolidated by decades of experience. To be transplanted, cord blood units must contain an adequate number of hematopoietic progenitors. Those units that do not fulfill the established cell thresholds for transplantation were not stored or provided donor consent and cannot be destined for additional clinical and research uses. According to the aforementioned Italian regulation, cord blood donors are given the ability to donate after meticulous medical counseling. Moreover, soon after collection, infectious screening tests are performed on the mother’s blood sample (human immunodeficiency virus, hepatitis B virus, hepatitis C virus, and syphilis) or on the unit (microbial test). Only units with proven negative tests were utilized for this study. The CB-PRP used in this study consisted of a pool of 15 units. Soon after collection, each unit was submitted to soft-spin centrifugation to obtain CB-PRP. The platelet count was normalized in all PRP at 1 × 10^9^/L platelets through hard-spin centrifugation of CB-PRP and subsequent removal of the platelet-poor plasma excess. Recovered CB-PRP was then stored at −80° C until microbial tests were proven negative. To avoid eventual disparities in growth factor concentrations among different units, 15 sterile CB-PRP samples were thawed, pooled, divided into 1-ml aliquots in sterile sealed tubes, and then stored again at −80° C until use (Fig 2), making the intervention homogeneous for all patients participating in the study. All necessary steps to prepare the CB-PRP occurred in sealed bags, and sterile connections were used to transfer the CB-PRP from 1 bag to another or to store aliquots of the CB-PRP pool. An additional microbial test was performed on the final product. The occurrence of sudden worsening of disease and/or bacterial infections and/or inflammatory reactions reasonably connected to the CB-PRP treatment in 2 consecutive patients’ eyes would have constituted a stopping rule for the study.

The surgical approach was 23-gauge (G) high-speed pars plana vitrectomy (10,000 cuts/minute). The Constellation Vision System (Alcon Laboratories, Inc.) was used for all surgeries. All patients received peribulbar anesthesia 30 minutes before surgery (10 ml of ropivacaine combined with 300 IU of hyaluronidase). Three valved cannulas were introduced at 2 o'clock and 11 o’clock for the service sclerotomies and in the inferior temporal quadrant for infusion at 3.5 mm from the sclero-corneal limbus. All eyes received detachment and removal of the posterior hyaloid membrane if not separated from the retinal layers below. Through a 41-G cannula, a subretinal injection of 0.5 ml of CB-PRP (CB-PRP) was performed below the retinal space in the inferonasal quadrant (Video 1). Afterward, complete peripheral vitrectomy was performed. Peripheral retinal photocoagulation was performed if any retinal tears, holes, or rhegmatogenous degenerations were observed. Fluid–air exchange was performed at the end of the surgery. After cannula removal, sclerotomies were checked, and bipolar diathermy or a single 8.0 nonresorbable polypropylene removable suture was used if necessary.^17^ After surgery, patients were recommended to maintain a supine position for several hours for 3 days.

In all enrolled patients in this study, a complete ophthalmological examination that included ETDRS BCVA assessment, anterior segment biomicroscopy, direct and indirect ophthalmoscopy, intraocular pressure measurement, OCT, and OCTA (Solix, Optovue) was performed at baseline and at 1, 3, 6, and 12 months. Two independent medical retina experts, using semiautomated software, calculated the atrophic areas using OCTA. The outline demarcation of GA in the device was allowed only on OCTA images. We combined the information of the best GA visualization in en face scan and OCTA image to outline the area of atrophy in OCTA. Regarding the GA area measurement, we decided to use en face images based on OCTA (the consensus agreement by Cohen coefficient was considered suitable only if >  0.9), which was recently demonstrated to be as reliable for fundus autofluorescence and en face structural OCT at the choroidal and scleral levels.^18^^,^^19^ The growth rate measurement of GA was in mm^2^. The following additional assessments were performed at baseline, 6 and 12 months: focal electroretinogram (f-ERG) and flicker visual evoked potentials (f-VEP).^20^^,^^21^ All the clinical morpho-functional data were also collected from the untreated eye and used as control data. To ensure the best safety for the patients, the first 5 eyes were temporally separated by a minimum of 20 days each to carefully observe the postsurgical evolution.

### Statistical Analysis

Statistical analysis was performed using SPSS software, version 26 (IBM SPSS Statistics), and Prism 8 software, version 8.2.1 (GraphPad Software). Quantitative variables were expressed as the mean and standard deviation. Two-way repeated-measures analysis of variance (ANOVA) was performed to test differences between treated and untreated eyes at baseline, 1, 3, 6, and 12 months and the interaction of treatment with time on BCVA, central macular thickness (CMT), and atrophy area in en face scans. Central macular thickness equal to center point measurements or central 1-mm measurements was automatically measured by the device, calibrated between the inner limiting membrane and ellipsoid zone in the macula region centered on the fovea. Post hoc analyses were performed by Tukey’s test. For f-ERG and f-VEP analysis, comparison of response amplitudes was performed using the *t* test for paired samples. A *P* value < 0.05 was considered statistically significant.

## Results

**Figure 3:**
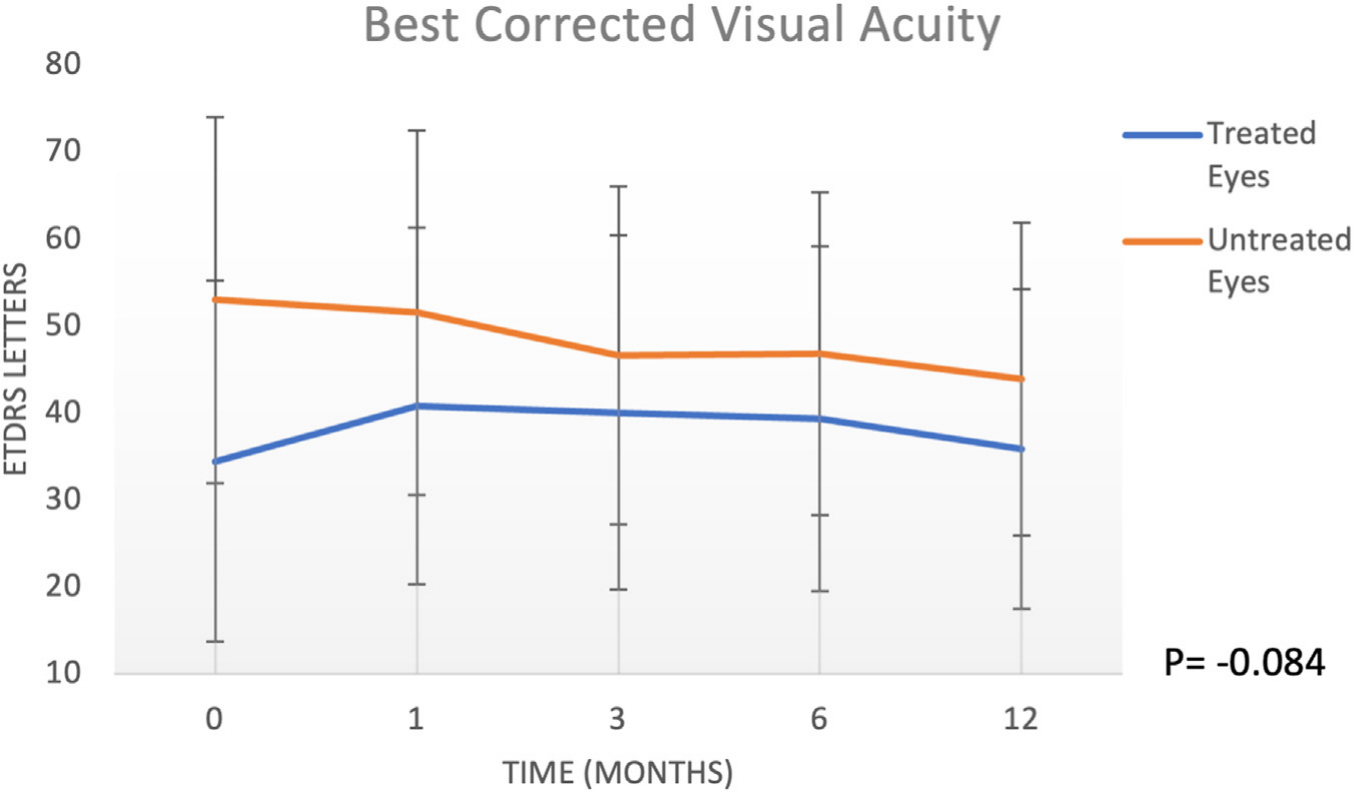
Best-corrected visual acuity using the scale from the EDTRS. Treated and untreated (fellow) eyes at 1, 3, 6, and 12 months of follow-up are reported. Error bars represent the standard deviation.

The patients’ mean age at surgery was 74.61 ± 6.71 years. The BCVA in the treated group was 34.46 ± 20.8 ETDRS at baseline, 40.84 ± 20.52 at 1 month, 40.07 ± 20.34 at 3 months, 39.38 ± 19.84 at 6 months, and 35.84 ± 18.38 at 12 months. In the untreated group, the BCVA was 53 ± 21.1 ETDRS letters at baseline, 51.54 ± 20.99 at 1 month, 46.62 ± 19.47 at 3 months, 46.85 ± 18.58 at 6 months, and 43.92 ± 17.97 at 12 months. Two-way ANOVA for visual acuity showed no effect of eye or time (*P* = 0.084, Fig 3).

**Figure 4:**
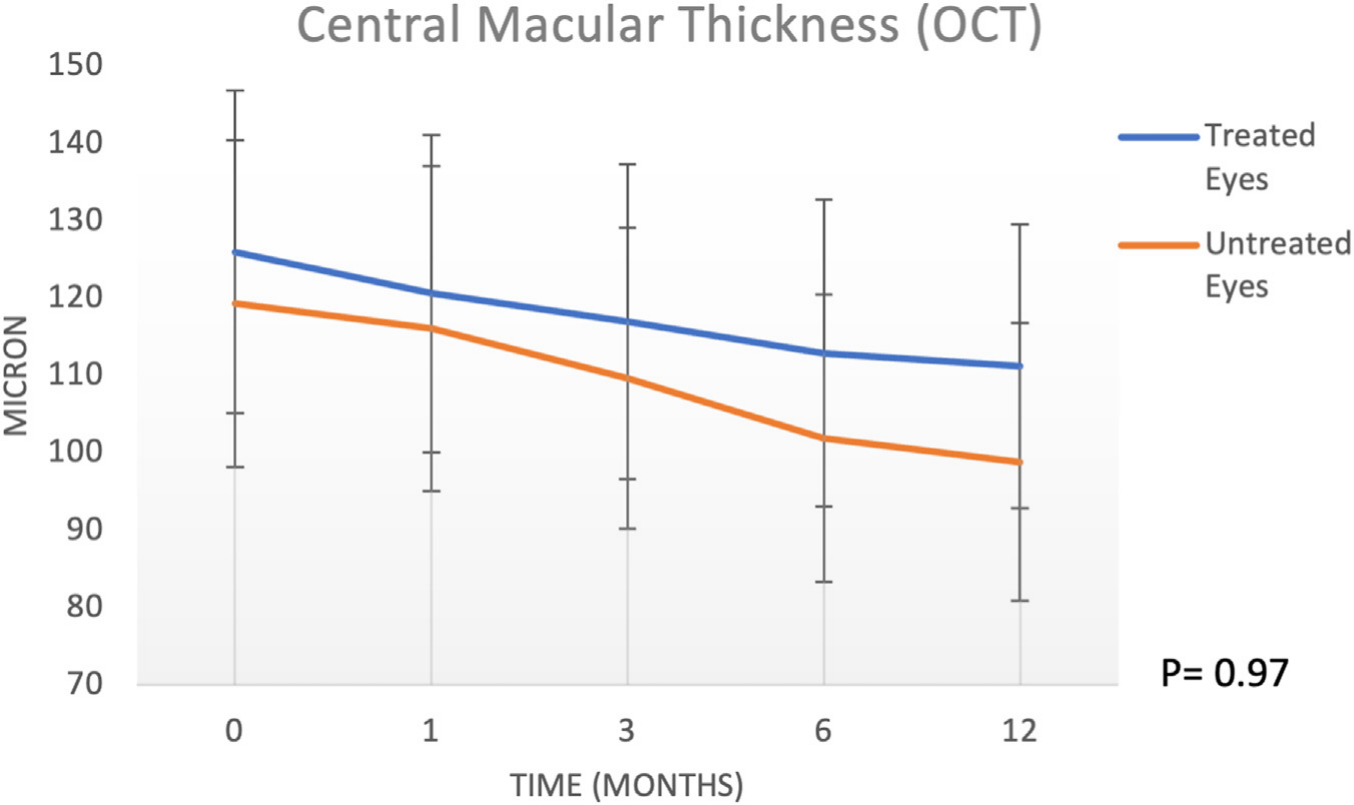
Central macular thickness (foveal) of treated and untreated (fellow) eyes at 1,3, 6, and 12 months of follow-up. These data were obtained using OCT (Solix, Optovue). Error bars represent the standard deviation.

**Figure 5:**
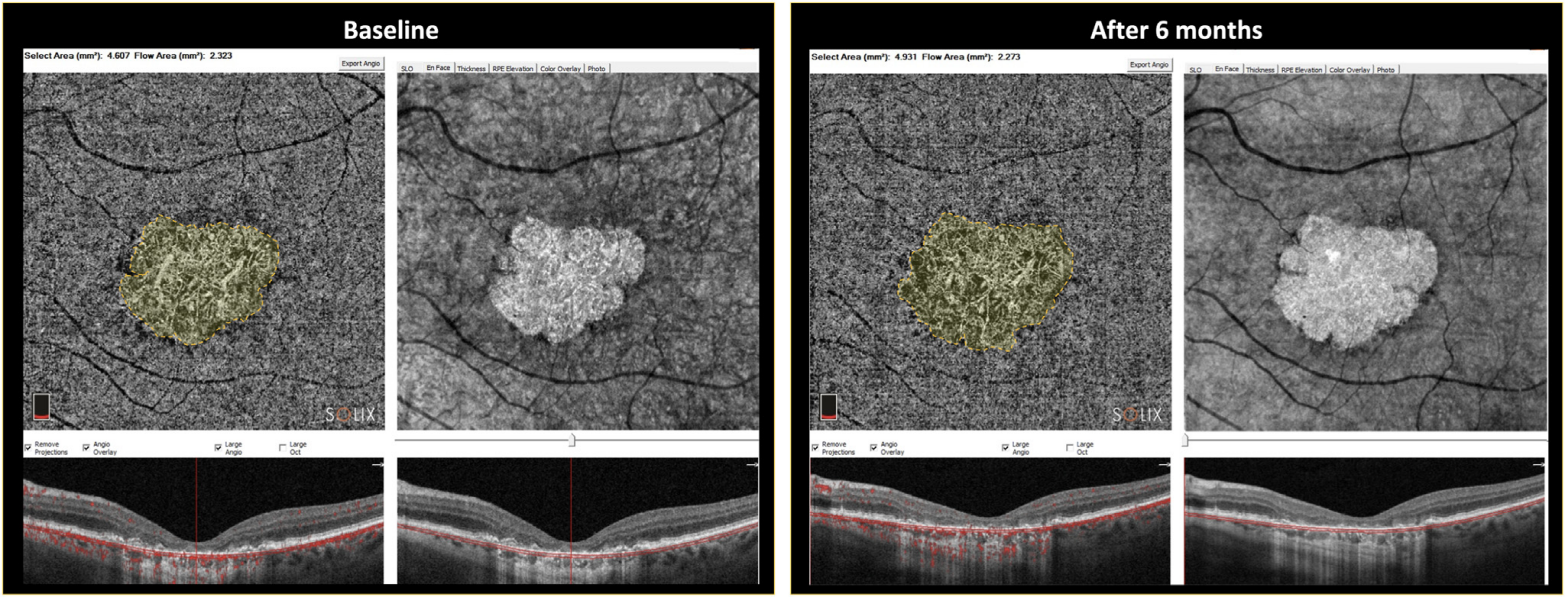
Semiautomated assessment image of 1 patient’s treated eye. Semiautomated assessment performed by 2 imaging experts (consensus agreement by Cohen coefficient was considered suitable only if > 0.9). The outline demarcation of geographic area was the result of the best visualization of atrophy in en face scan and OCT angiography image. Baseline and after 6 months images show the growth of geographic atrophy (mm^2^) quantified in the selected area tool.

The CMT in the treated group was 120.54 ± 52.16 μm at baseline, 119.31 ± 50.91 at 1 month, 118 ± 49.99 at 3 months, 117.46 ± 53.91 at 6 months and 111.23 ± 52.37 at 12 months. In the untreated group*,* the CMT was 119.31 ± 58.52 μm at baseline, 118.92 ± 56.16 at 1 month, 117.46 ± 58.52 at 3 months, 107.07 ± 54.97 at 6 months and 98.85 ± 54.55 at 12 months. Two-way ANOVA for CMT did not show a significant intereye difference at 12 months (*P =* 0.97; Fig 4). However, Tukey’s test showed a significant difference at 6 months (*P* < 0.05) between treated and untreated eyes, with CMT thinner in control eyes than in treated eyes. For OCTA atrophic area calculation, the Cohen κ index of agreement between the 2 expert and independent analyzers was > 90% (Fig 5). The atrophic area by means of en face scans in the treated group was 15.16 ± 9.92 mm^2^ at baseline, 15.18 ± 9.92 at 1 month, 15.74 ± 9.83 at 3 months, 16.98 ± 9.88 at 6 months, and 17.35 ± 9.78 at 12 months. In the untreated group, the atrophic area was 8.47 ± 4.52 mm^2^ at baseline, 9.47 ± 4.85 at 1 month, 10 ± 4.86 at 3 months, 11.17 ± 4.91 at 6 months, and 11.59 ± 4.75 at 12 months (Table 1). We observed a significant atrophic enlargement in both treated and untreated groups (*P* < 0.001) at 12 months.^22^

**Table 1:** Data and Changes of Best-Corrected Visual Acuity, Central Macular Thickness, and Area of Atrophy in Treated and Untreated Eyes Treated eyes

	Baseline	1 month	3 months	6 months	12 months	*P* Value (2-Way ANOVA)
BCVA (ETDRS letters)	34.46 ± 20.8	40.84 ± 20.52	40.07 ± 20.34	39.38 ± 19.84	35.84 ± 18.38	*P* = 0.06
CMT in OCT (μm)	120.54 ± 52.16	119.31 ± 50.91	118 ± 49.99	117.46 ± 53.91	111.23 ± 52.37	*P* = 0.03
Atrophy area on en face OCT (mm^2^)	15.16 ± 9.92	15.18 ± 9.92	15.74 ± 9.83	16.98 ± 9.88	17.35 ± 9.78	*P* < 0.0001
Atrophic area progression (SQRT∗ – mm/yr)	0.2709					

Untreated eyes						
	Baseline	1 month	3 months	6 months	12 months	*P* Value (2-Way ANOVA)
BCVA (ETDRS letters)	53 ± 21.1	51.54 ± 20.99	46.62 ± 19.47	46.85 ± 18.58	43.92 ± 17.97	*P* = 0.02
CMT in OCT (μm)	119.31 ± 58.52	118.92 ± 56.16	117.46 ± 58.52	107.07 ± 54.97	98.85 ± 54.55	*P* < 0.001
Atrophy area on en face OCT (mm^2^)	8.47 ± 4.52	9.47 ± 4.85	10.00 ± 4.86	11.17 ± 4.91	11.59 ± 4.75	*P* < 0.0001
Atrophic area progression (SQRT∗ – mm/yr)	0.4946					

ANOVA = analysis of variance; BCVA = best-corrected visual acuity; CMT = central macular thickness using OCT; SQRT = square root transformation.

**Figure 6:**
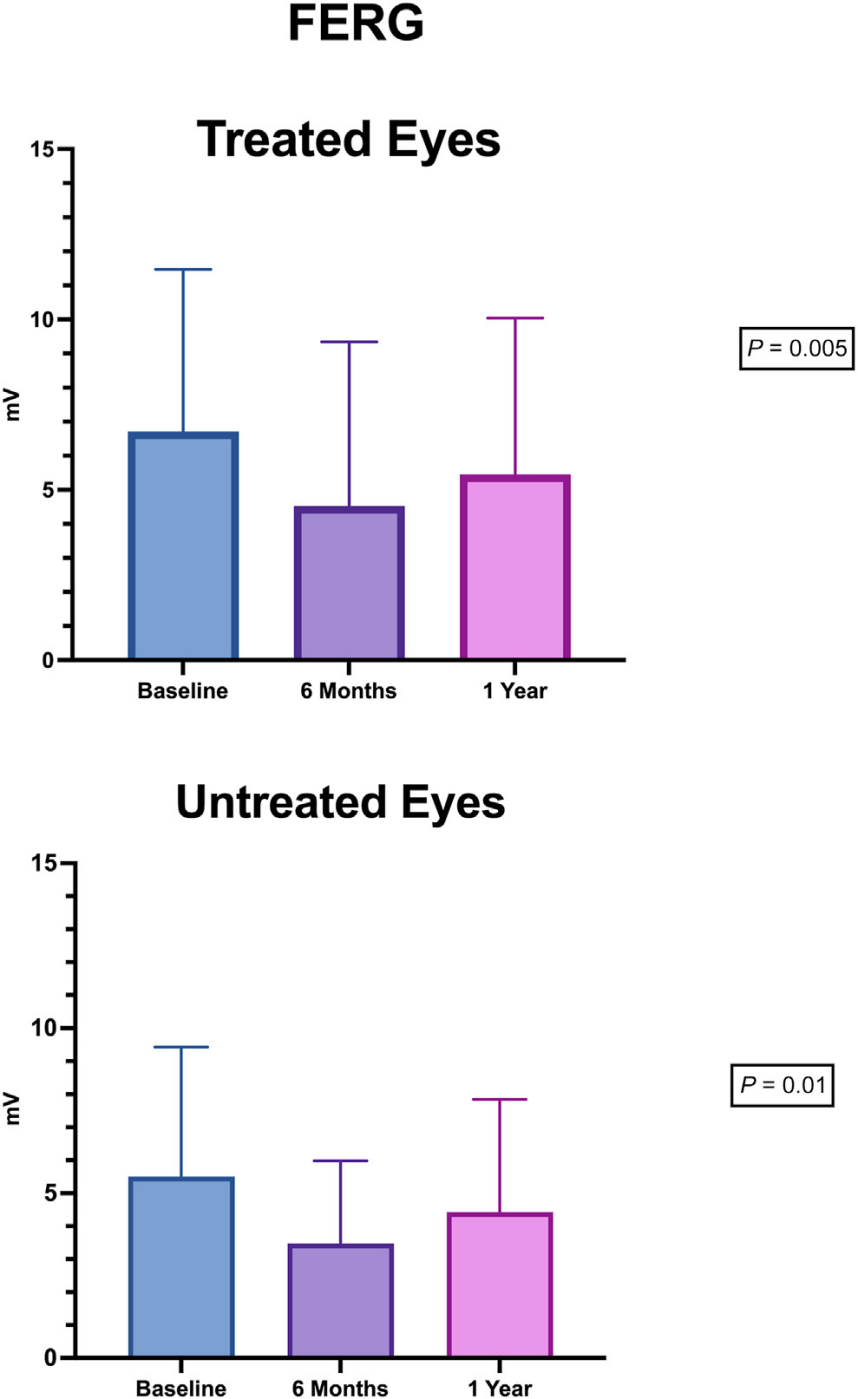
Focal electroretinogram (FERG) amplitude during the follow-up in treated and untreated eyes.

**Figure 7:**
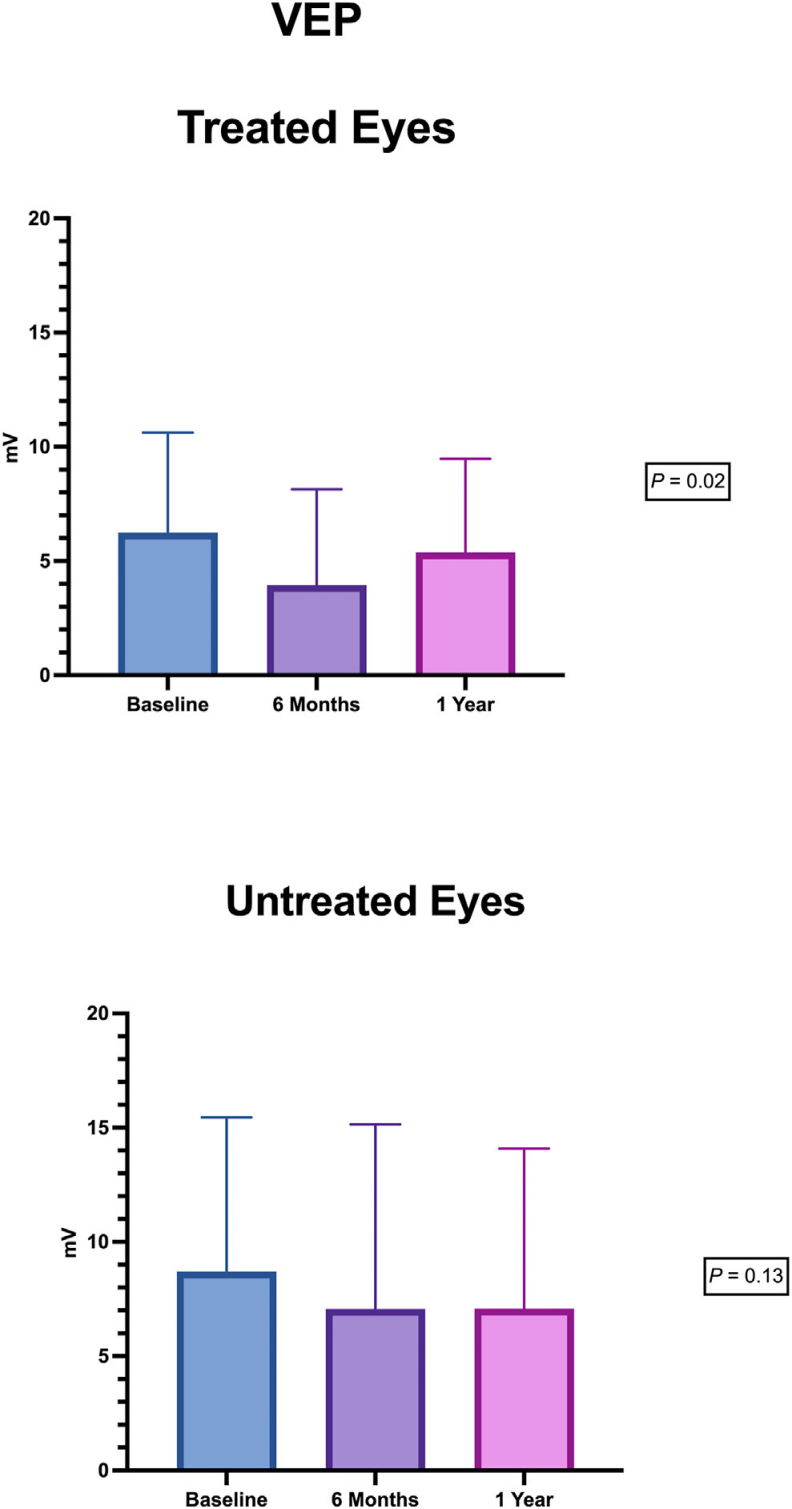
Flicker visual evoked potential (f-VEP) amplitude during the follow-up in treated and untreated eyes.

Mean f-ERG and f-VEP amplitudes showed a decrease in amplitude in both treated and fellow eyes at 6 months compared with baseline. The changes were significant in the paired *t* test (*P* < 0.01). These differences were not observable anymore at 12 months (f-ERG treated, *P* = 0.005 vs. untreated, *P* = 0.01; f-PEV treated, *P* = 0.02 vs. untreated, *P* = 0.13; Figure 6, Figure 7). No inflammatory reaction, endophthalmitis, retinal detachment, uveitis, or other complications due to the subretinal injection of CB-PRP were observed during the follow-up.

## Discussion

In the last decade, d-AMD-related GA research has focused on anti-inflammatory agents and complement inhibitors. Eculizumab (monoclonal antibody that targets complement protein C5 [C5]), lampalizumab (a humanized mAb directed against factor D, key regulator of the alternate pathway of complement activation), sirolimus (blocks the activation of the cell-cycle-specific kinase) and tesidolumab (targets and binds to C5,) did not achieve significant reductions in GA progression.^23^, ^24^, ^25^, ^26^ However, there are high expectations for zimura (a C5 inhibitor), pegcetacoplan (binds to C3 and inhibits its activation), and risuteganib (blocks oxidative stress in RPE cells). Inhibition of the complement pathway in the pathogenesis of AMD was assessed by avacincaptad pegol (Zimura, IVERIC bio Inc.). Study results showed that avacincaptad pegol was generally well tolerated after monthly administration and led to a significant reduction of GA growth in eyes with AMD over a 12-month period compared with their corresponding sham cohorts.^5^ The FILLY study of a monthly or every other month pegcetacoplan injection found a decrease in GA growth rates of 29% (*P* = 0.008) and 20% (*P* = 0.067), respectively. However, there were no significant differences in BCVA results across the groups.^27^

Comparing the monthly pegcetacoplan (Syfovre) intravitreal injection to our subretinal CB-PPR injection, the change in GA lesion size from baseline up to 12 months was 0.26 mm and 0.27 mm (square root transformation), respectively.^28^

In comparison with avacincaptad pegol (Zimura), the mean rate of GA growth (square root transformation) was 27.4% for the 2-mg cohort and 27.8% for the 4-mg cohort compared with their corresponding sham cohorts.^5^

However, both treatments (pegcetacoplan and avacincaptad pegol) were intravitreal and monthly administered injections, and the reported results were at 12 months, differently from our study that supposed a single subretinal injection of CB-PRP.

Shaw et al^29^ reported that 1 mg of risuteganib resulted in an increase of ≥ 8 letters in 48% of the patients, compared with 7.1% in patients receiving a placebo, which was statistically significant.

An important breakthrough was that, on February 2023 and August 2023, the Food and Drug Administration approved intravitreal pegcetacoplan and avacincaptad pegol, respectively, as the only and first treatments for GA.^7^^,^^8^ This event represents a radical change in the management and treatment of d-AMD-related GA.

Another important chapter is represented by neuroprotective agents, aimed at reducing apoptosis and eventually preventing the progression of GA in AMD, with promising preclinical studies.^9^^,^^30^^,^^31^ After this thread, the current pilot study was designed to evaluate subretinal CB-PRP as a neuroprotective therapeutic strategy for GA in d-AMD. This hemoderivate is well known in several fields of medicine, such as gynecology and reproductive medicine, dermatology and cosmetology, sports medicine, dentistry, neurology and neurosurgery, and orthopedics.^13^ Due to growth factors, including platelet-derived growth factor, insulin-like growth factor-1, epidermal growth factor, hepatocyte growth factor, transforming growth factor, basic fibroblast growth factor, and VEGF, CB-PRP helps to control all stages of the healing process and regeneration of damaged tissues. In ophthalmology, CB-PRP is mostly used in the treatment of corneal ulcers, dry eye syndrome, and corneal surface regeneration.^32^, ^33^, ^34^, ^35^ Cord blood PRP has excellent regenerative properties owing to its high concentration of platelets that release biologically active ingredients and growth factors from their granules in the activation process, although the potential is still not fully understood.^13^ Conditions such as persistent epithelial defects, neurotrophic keratitis, ocular surface chemical injury, postkeratoplasty persistent epithelial defect, and postlaser epithelial keratomileusis support have all been successfully treated with CB-PRP eye drops.^36^, ^37^, ^38^

**Figure 8:**
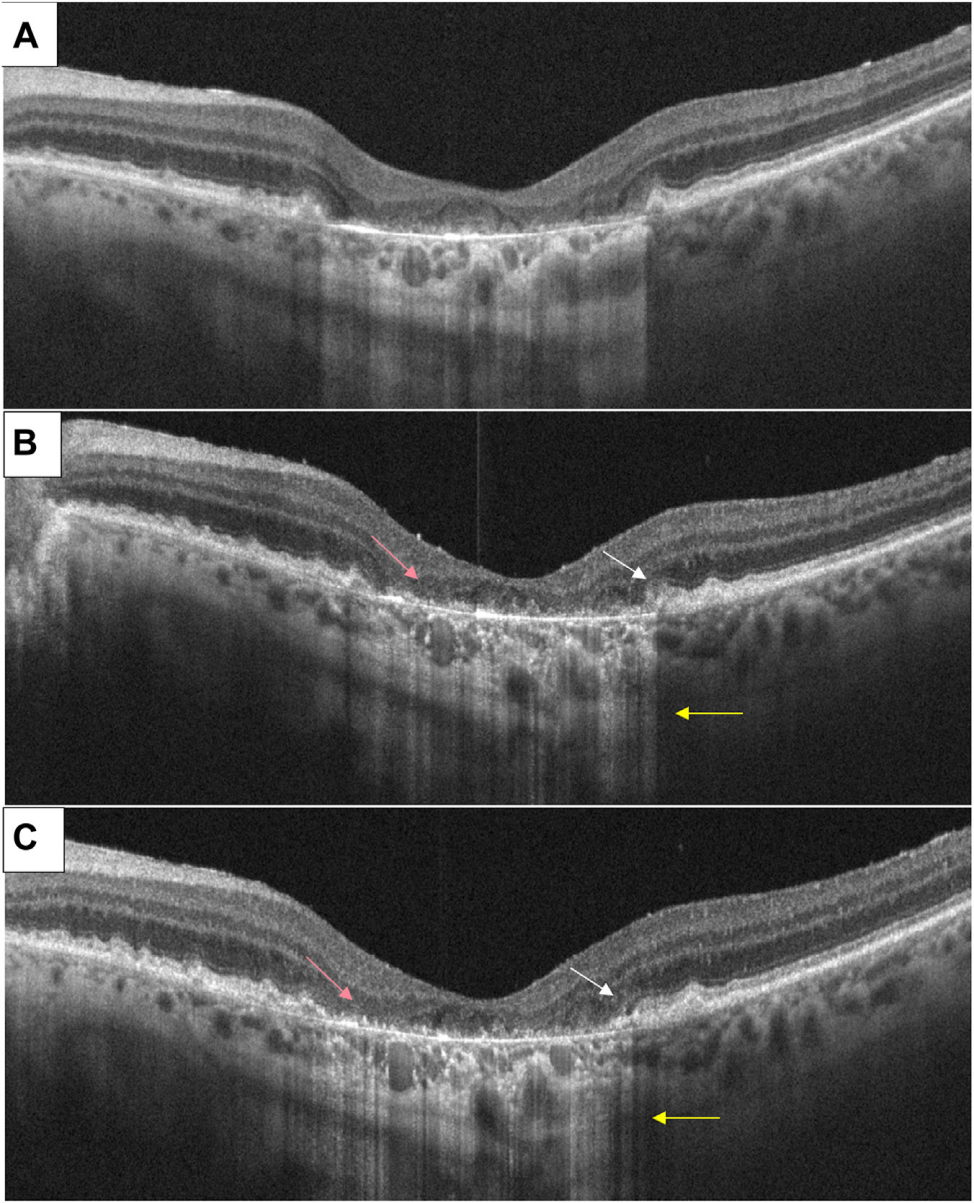
Structural OCT B-scan characteristics before cord blood platelet-rich plasma subretinal injection (**A**) and at 3 months (**B**) and 6 months (**C**). White arrows show retinal pigment epithelium (RPE) remodeling at the edge of the retinal atrophic area at 3 and 6 months. Yellow arrows indicate back scattering reduction at 3 and 6 months. Pink arrows show RPE “granulation” at 3 and 6 months (uncertain meaning).

Our findings showed that, after the subretinal injections of CB-PRP, none of the treated eyes showed any side effect at 12-month follow-up. We have also recorded a possible signal of efficacy on the rate of growth of the atrophic area in treated eyes compared with fellow control eyes, but these findings deserve additional and larger study to be confirmed or confuted. As shown in Figure 8, we observed a likely remodeling of the RPE at the margins of the atrophic area of the retina at 3 and 6 months after subretinal injection of CB-PRP. This effect could be attributed to higher and different concentrations of growth factors, such as epidermal growth factor, transforming growth factor, transforming growth factor-2, fibroblast growth factor, platelet-derived growth factor, VEGF, nerve growth factor, interleukin-1β, interleukin-4, interleukin-6, interleukin-10, and interleukin-13, compared with other adult blood-derived preparations, with lower concentrations of insulin-like growth factor-1, insulin-like growth factor-2, and transforming growth factor-1.^10^ Di Marco et al^14^ supported the idea that retinal neurodegenerative processes might be successfully mitigated using eye drops based on CB-PRP. In rat eyes that received CB-PRP drops, the function was partially maintained, and the retinal morphology appeared to be better compared with the control group. Neurotrophic factors and cytokines contained in CB-PRP could represent an evolution in this field due to their greater power to promote the expansion of mesenchymal stromal cells in culture, resulting in faster healing. Positive clinical outcomes have been seen in several fields, such as oral lesions, diabetic-related foot ulcers, or perianal fistulae.^39^, ^40^, ^41^

To the best of our knowledge, the efficacy/safety of subretinal injection of CB-PRP for the treatment of AMD has not yet been investigated. To optimize the efficacy and bypass the inner blood-retinal barrier, we chose subretinal administration, which is already used for gene therapy or cell-based therapies. The administration procedure was shown to be very safe and without any complications in our study population. In our patients, the site of the injection was chosen in the inferonasal quadrant to avoid macular retinal tears at the edge of the atrophic macular area during retinal bleb formation. It is well known that the paracrine effect of neurotrophic factors allows the latter to have their effects, even if they are administered far from the site of the target tissue.^42^

Regarding the results of BCVA, the main purpose of this study was to report the safety of sub retinal injection of CB-PRP. In treated eyes we did not observe a decline or significantly worsening of the BCVA at 12 months. We speculate that the post-treatment gain of some ETDRS letters in *treated group*, could be related to the surgery itself. In fact, complete vitrectomy was performed in all eyes so that a slight improvement in BCVA could be expected due to vitreous removal, rendering the ocular media more transparent. On the other hand, the BCVA decline in the *untreated group* was comparable to that reported in natural history studies regarding GA.^43^

Regarding the structural changes, in our patients, there was no statistically significant difference in CMT between treated and untreated eyes at 12 months (*P* = 0.97). However, at 6 months, the CMT of untreated eyes was thinner than that of treated eyes, suggesting a possible temporal protective effect of the CB-PRP on central retinal degeneration. Clearly, a lager cohort of patients, which was beyond the scope of this pilot study, would be required to confirm this finding. For GA area measurement, we used en face OCT at the choroidal and scleral levels, which was recently demonstrated to be as reliable as fundus autofluorescence.^18^ An interesting finding is the difference in the progression of the macular atrophic area between treated and untreated eyes at 1 to 3 months postoperatively. Treated eyes showed a slower rate of GA enlargement than untreated fellow eyes at months 1 and 3 (*P* = 0.06 and *P* = 0.002 respectively compared with untreated *P* < 0.001). One concept worth emphasizing is that we have treated the worst eye, and GA enlargement in those eyes could already have a low rate of progression due to the more advanced stage of the disease.

The results of functional examinations (mean f-ERG and f-VEP) showed a decrease in amplitude in both treated and fellow eyes at 6 months compared with baseline. The changes were significant in the paired *t* test (*P* < 0.01). Remarkably, those functional examinations did not show a significant difference at 12 months (f-FERG treated, *P* = 0.005 vs. untreated, *P* = 0.01; f-PEV treated, *P* = 0.02 vs. untreated, *P* = 0.13). This f-ERG amplitude reduction during the first 6 months in treated eyes is in line with the effect of multiple growth factors on retinal function as reported by Gargini et al^44^ Similarly, protection of photoreceptors from light damage is linked to changes in growth factor expression and f-ERG characteristics. For this reason, the reduction in f-ERG amplitude could reflect a temporary protective effect on photoreceptors from damage through control of retinal sensitivity to light.^14^ We speculate that the beneficial effects of CB-PRP observed in our study could have a limited duration, likely linked to the half-life of the growth factors contained in CB-PRP. Longer follow-ups are needed to confirm this hypothesis.

In fact, on the other hand, we are not able to explain the reason why this f-ERG amplitude reduction during the first 6 months was also registered in the untreated eyes.

### Limitations and Strengths

Our study had several limitations. First, it was a single-center, open-label and nonrandomized trial. Second, the number of patients enrolled in our pilot study was very limited, although it had sufficient power to demonstrate the safety of CB-PRP used as subretinal injection. Also, the short follow-up and the lack of repeated injections (due to the method of administration) were great limitations to analyze a possible real effect of CB-PRP in d-AMD. Finally, the large variability among each group in BCVA and GA lesion area, and lack of randomization, are further limitations and potential sources of bias in our study.

On the other hand, 1 of the strengths was the production of CB-PRP we used in this study. Actually, it was created starting from 15 placenta donations (*pool*). In this way, each CB-PRP aliquot contained the same profile and amount of neurotrophic factors and cytokines, resolving the issue regarding the different neurotrophic factor concentrations that different placentas can naturally have.

In summary, this study provided for the first time, to our knowledge, proof of the principle that subretinal injection of CB-PRP could be safely used in GA associated with dry-AMD. No side effects and/or retinal or ocular damage was observed during the follow-up. Even if our findings support a potential role of the CB-PRP in the GA management, a larger cohort of patients will be needed to confirm or confute our results. The exploration of other methods of administration would be desirable as well.

## Supplementary Data

Video 1Subretinal injection of CB-PRP. Twenty-three-gauge vitrectomy is shown, and CB-PRP subretinal injection (41-G subretinal needle) in the nasal retinal quadrant is performed.
